# Differential RISC association of endogenous human microRNAs predicts their inhibitory potential

**DOI:** 10.1093/nar/gkt1393

**Published:** 2014-01-23

**Authors:** Omar Flores, Edward M. Kennedy, Rebecca L. Skalsky, Bryan R. Cullen

**Affiliations:** Department of Molecular Genetics & Microbiology and Center for Virology, Duke University Medical Center, Durham, NC 27710, USA

## Abstract

It has previously been assumed that the generally high stability of microRNAs (miRNAs) reflects their tight association with Argonaute (Ago) proteins, essential components of the RNA-induced silencing complex (RISC). However, recent data have suggested that the majority of mature miRNAs are not, in fact, Ago associated. Here, we demonstrate that endogenous human miRNAs vary widely, by >100-fold, in their level of RISC association and show that the level of Ago binding is a better indicator of inhibitory potential than is the total level of miRNA expression. While miRNAs of closely similar sequence showed comparable levels of RISC association in the same cell line, these varied between different cell types. Moreover, the level of RISC association could be modulated by overexpression of complementary target mRNAs. Together, these data indicate that the level of RISC association of a given endogenous miRNA is regulated by the available RNA targetome and predicts miRNA function.

## INTRODUCTION

The large majority of human microRNAs (miRNAs) are initially transcribed by RNA polymerase II as part of a long pri-miRNA precursor that is processed by Drosha, in the nucleus, and Dicer, in the cytoplasm, to generate the miRNA duplex intermediate (1). One strand of this ∼22-bp dsRNA is then loaded into the RNA-induced silencing complex (RISC), which consists minimally of one of the four human Argonaute (Ago) proteins Ago1 to Ago4. Loading into RISC leads to the release of the passenger strand into the cytoplasm, where it is degraded by exonucleases.

Because the non–RISC-associated passenger strand is highly labile, it has been suggested that the contrasting high stability of miRNAs reflects their RISC association (2–5) and, therefore, that sequencing of the total cellular miRNA population accurately reflects the RISC-associated miRNA population. This assumption has been recently challenged by two manuscripts that quantified the Ago association of endogenous miRNAs or exogenous small interfering RNAs in cultured cells and reported that only a small proportion, perhaps <10%, of these small RNAs are actually in RISC (6,7). In contrast, a substantial percentage of the miRNA pool appeared to be bound to mRNAs in an Ago-unbound form. If miRNAs are indeed found *in vivo* in a form that is not bound to the RISC effector, then this raises the possibility that the RISC association, and hence inhibitory potential, of miRNAs is differentially regulated. Here, we demonstrate that this is indeed the case and present evidence that the level of Ago association of individual miRNAs is influenced by the availability and degree of complementarity of mRNA target species.

## MATERIALS AND METHODS

### Cell culture

Human 293 cells were propagated in Dulbecco modified Eagle medium (DMEM) supplemented with 10% fetal bovine serum (FBS). C8166 and A549 cells were propagated in Roswell Park Memorial Institute medium (RPMI) supplemented with 10% FBS. LCLs were propagated in in RPMI supplemented with 15% FBS and 1% gentamicin (Gibco). SH-SY5Y cells were maintained in a 1:1 mixture of Minimal Essential Medium Eagle (MEME) and Ham’s F-12 medium with 10% FBS and 1% non-essential amino acids.

### RNA recovery, sequencing and bioinformatics

RISC-bound miRNAs were isolated by immunoprecipitation using one of two different monoclonal antibodies specific for the human Ago proteins: 2A8 (diagenode) or ab57113 (Abcam). 2A8, raised against Ago2 residues 47–879 (8), has been previously reported to recognize all four human Ago proteins, while ab57113, raised against Ago2 residues 483 to 859, was found to effectively bind human Ago1, Ago2 and Ago3 in our laboratory (data not shown). Although the high level of protein sequence conservation suggests that ab57113 likely also recognizes human Ago4, which contributes only a minor part of the total Ago pool, we were unable to confirm this as we were unable to express Ago4.

A 2-ml cell pellet was lysed in lysis buffer [50 mM HEPES pH 7.5, 150 mM KCl, 2 mM EDTA, 1 mM NaF, 0.5% (vol/vol) NP-40, 0.5 mM dithiothreitol (DTT), protease inhibitors] and was then centrifuged to remove insoluble particulates. The lysate was then incubated on a rotator overnight at 4°C with protein G beads loaded with the anti-Ago antibody. The beads were then washed 10 times with NT2 buffer (50 mM Tris-HCl pH 7.4, 150 mM NaCl, 1 mM MgCl2, 0.05% NP-40). The washed beads were then incubated in NT2 buffer in the presence of proteinase K prior to extraction using acid–phenol. The aqueous phase was then used to isolate small RNAs (<200 nt) using a mirVana kit (Ambion). Typically, this resulted in the recovery of ∼250 ng of total small RNAs from each sample. Total RNA was isolated using acid–phenol, and the aqueous phase was then used to isolate small RNAs (<200 nt) using a mirVana kit. The RNA immunoprecipitation (RIP) and Total RNA cDNA library was constructed essentially as described previously (9) by using an Illumina TruSeq small-RNA kit prior to sequencing with an Illumina HiSeq 2000 system. The resulting reads were then processed using the FASTX-Toolkit and were aligned to the human genome (NCBI, Hg19) and human miRNA library (miRBase19), using Bowtie (10). Reads of at least 15 nt were retained if they mapped to the indicated library with 0 mismatch.

Analysis of tailed reads was completed using custom Perl scripts written in house. Briefly, deep sequencing reads obtained from the above pipeline were aligned with Bowtie (10) to miRBase19 hairpins with no mismatches, and reads which were unaligned were retained. These reads were confirmed to have a discrete 5′ prime start site and were then assigned to the human miRNA of closest length with a complete, perfect match along the length of the mature miRNA. Then the sequence 3′ of the potentially tailed, mature miRNA was checked against all pre-miRNA hairpins capable of generating it, and miRNAs were designated tailed if the 3′ sequence extension did not align to any pre-miRNA hairpin.

### qRT-PCR

RNA isolated as described above was also used to determine miRNA levels using TaqMan microRNA quantitative reverse transcription PCR (qRT-PCR) assays (Applied Biosystems). Assays were performed in triplicate, and miRNA values were normalized to the level of miR-138, which was found to have neither selective loading nor exclusion from RISC. The assay identification numbers (IDs) are 002284 for miR-138-5p, 000398 for miR-22-3p, 000431 for miR-92a-3p, 002253 for miR-101-3p and 000497 for miR-197-3p.

### Luciferase assays

Sensor vectors were generated using psiCHECK-2 (Promega), which contains both RLuc and FLuc. Three full-length complementary synthetic binding sites, with mismatches at nucleotides 10 and 11 of the miRNA, for the miRNA of interest were inserted using NotI and XhoI sites present in the 3′ untranslated region (3′ UTR) of RLuc. Sensor constructs (50 ng) were transfected into a 24-well plate seeded with 293 or A549 cells using Lipofectamine 2000 (Invitrogen) according to the manufacturer’s protocol. Cells were harvested 24 h later, and RLuc and FLuc (internal control) levels then determined using Dual Luciferase-reporter assays (Promega), according to the manufacturer’s protocol.

### miRNA loading assay

Sensor constructs specific for miR-22, miR-101 and miR-197, similar to the ones used for indicator assays, were transfected into 293 cells using Lipofectamine 2000 (Invitrogen) according to the manufacturer’s protocol. We used 40 µg of each sensor construct per 150 mm tissue culture plate. Cells were harvested 72 h later. Total RNA and RISC associated RNA was then isolated as described above and miRNA levels determined by TaqMan microRNA assay (Applied Biosystems).

## RESULTS

### Differential RISC association of endogenous microRNAs

**Figure 1.**
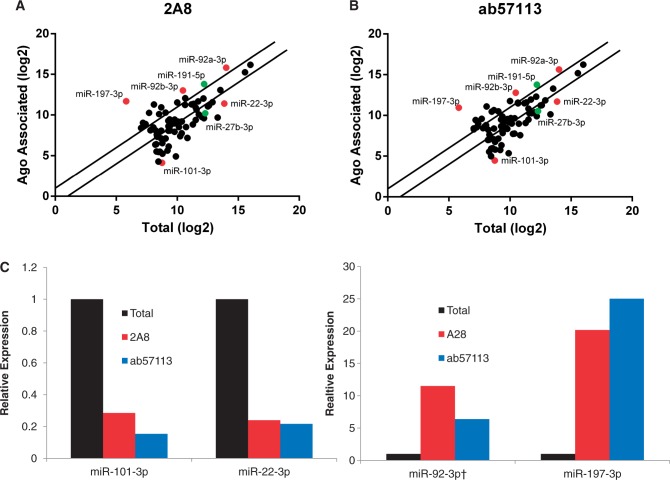
Differential RISC association of endogenous miRNAs in 293 cells. (**A** and **B**) Illumina deep sequencing was performed for either RISC-associated miRNAs, isolated by immunoprecipitation using either the diagenode 2A8 or Abcam ab57113 pan-Ago specific monoclonal antibody, or Total small RNAs (≤200 nt). Raw reads were aligned to mature human miRNAs and normalized to total miRNA-aligned reads. Individual miRNAs that represented >0.1% of either miRNA population were then raised to log base 2 and plotted as percent miRNA reads in the eluate versus percent reads in the total miRNA pool. Lines indicate a 2-fold difference from the mean. miRNAs highlighted in grey were verified by qRT-PCR (see below), while miRNAs indicated by name were used for functional assays. (**C**) qRT-PCR was performed using Taqman probes for miR-101-3p, miR-22-3p, miR92–3p and miR-197-3p using the same RNA preparations used for deep sequencing in panels A and B. (†) Note that the Taqman probe used to measure miR-92a-3p levels will also detect miR-92b-3p. However, both of these closely related miRNAs are predicted to be equally overrepresented in RISC (panels A and B).

To examine whether different species of endogenous miRNAs show different levels of RISC association in cultured human cells, we used deep sequencing to compare the relative level of expression of human miRNAs in the total small (<200 nt) RNA population found in 293 cells as well as in the RISC-associated small RNA population isolated by immunoprecipitation using the pan-Ago monoclonal antibody 2A8 (8). This analysis identified 1153 detectable human mature miRNAs or miRNA passenger strands (represented in miRBase v19), of which 76 were expressed at or above the level, ≥0.1% of the total miRNA population, proposed by others to represent the cut-off for functionally relevant miRNAs (11). As shown in Figure 1A, and detailed in Supplementary Table S1, we observed that several of these highly expressed miRNAs were either more highly RISC associated (e.g., miR-197-3p, miR-191-5p, miR-92a-3p and miR-92b-3p) or significantly less RISC associated (e.g., miR-22-3p, miR-27b-3p and miR-101-3p) than the average miRNA. In total, of the 76 distinct miRNA species found at levels representing >0.1% of the total and/or RISC-associated miRNA pool in 293 cells, 17 were found to be >2-fold more RISC associated than the average, while 34 were found to be <2-fold as RISC associated. The most highly RISC-associated endogenous miRNA, miR-197-3p, was found to be ∼59 times more RISC associated than the average miRNA, while the least RISC-associated miRNA, miR-1307-5p, was ∼31-fold less highly RISC associated than the average miRNA (Supplementary Table S1). This is a total difference of >1800-fold and implies, assuming that miR-197-3p is 100% in RISC, that <0.1% of the miR-1307-5p detected in these 293 cells is actually present in RISC. A similar picture also emerged when miRNAs representing <0.1% of the total miRNA pool were analysed (data not shown).

To confirm the reproducibility of this result, we repeated this experiment using a second, distinct pan-Ago monoclonal antibody, ab57113. This analysis revealed a strong correlation (Pearson correlation *r* = 0.99) between the relative level of a given miRNA detected by deep sequencing when RISC was immunoprecipitated using either the 2A8 or the ab57113 antibody, with no miRNA showing a ≥2-fold discrepancy between these two data sets (Supplementary Figure S1). In contrast, total miRNA levels, when compared with the RISC-associated miRNA levels recovered using either 2A8 (Figure 1A) or ab57113 (Figure 1B), exhibited a substantially lower degree of correlation (Pearson correlation *r* = −0.68), with no less than 51 out of the 76 highly expressed miRNAs analysed showing a >2-fold discrepancy in detection between these data sets (Figure 1A and B).

Several groups have reported that deep sequencing can substantially over- or undercount specific small RNAs due to intrinsic, sequence-specific differences in the ligation procedures used (12,13). While this seemed unlikely to explain the differential RISC association noted in Figure 1A and B, as the sequences of the miRNAs examined are the same in both the total and RISC-associated small RNA fractions, we nevertheless decided to use a second, distinct assay to measure RISC association. As shown in Figure 1C, we were able to fully confirm the preferential RISC association of miR-92a-3p/miR-92b-3p and miR-197-3p, and the weak RISC association of miR-101-3p and miR-22-3p, by qRT-PCR analysis. We note that analysis of the level of Ago protein recovered by the IP procedure used showed that ∼88% of the total Ago pool was solubilized and immunoprecipitated, with only ∼4% of the Ago pool remaining in the insoluble fraction and ∼9% remaining in the depleted lysate after immunoprecipitation (Supplementary Figure S2). Therefore, these differences in mRNA recovery do not arise from our inability to effectively immunoprecipitate miRNA-bound Ago proteins, and these data argue against the hypothesis that the observed discrepancy in miRNA loading reflects the existence of a pool of RISC that is resistant to immunoprecipitation.

**Figure 2.**
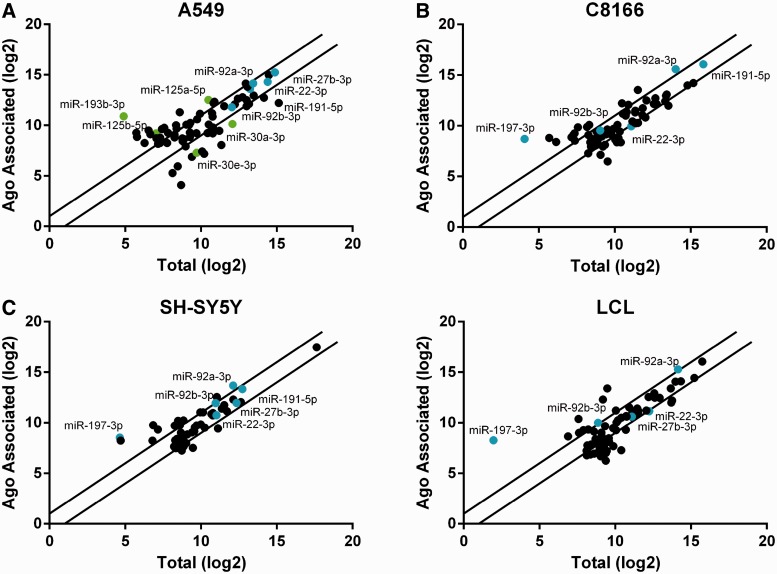
MiRNA distribution in various human cell types. (**A**) Illumina deep sequencing was performed using A549 cells for either RISC-associated miRNAs or Total miRNAs. Raw reads were aligned to mature human miRNAs and normalized to total aligned reads. Individual miRNAs representing >0.1% of either miRNA pool were then raised to log base 2 and plotted. Lines indicate a 2-fold difference in representation in the two libraries. miRNAs indicated by name were used in functional assays, or are highlighted to compare their RISC association with that seen in 293 cells. (**B**) miRNAs expressed in C8166 cells were analysed in the same manner as in panel A. miRNAs indicated by name showed differential RISC association in 293 cells. (**C**) miRNAs expressed in SH-SY5Ycells were analysed in the same manner as in panel A. (**D**) miRNAs expressed in the LCL were analysed in the same manner as in panel A.

We next asked whether the phenomenon of differential RISC association reported in Figure 1 was unique to 293 cells by analysing four other human cell lines derived from different human tissues. Analysis of A549 cells (lung), C8166 cells (T cells), SH-SY5Y cells (neuroblastoma) or LCL (B cells) in fact revealed that individual, highly expressed miRNAs were again over- or underrepresented in RISC, with differences of >100-fold in the relative level of RISC association being again observed (Figure 2 and see Supplementary Table S2 for a list of all 73 miRNAs analysed in A549 cells). A comparison with 293 cells showed that miR-197-3p was overrepresented in RISC in all three cell lines that express this miRNA. Conversely, the closely similar miR-92a-3p and miR-92b-3p were either both slightly overrepresented in RISC, e.g. in SH-SY5Y cells and the LCL, or were found at comparable levels in the RISC-associated and total miRNA pools, e.g., in A549 cells. Finally, miR-27-3p and miR-22-3p, which were underrepresented in RISC in 293 cells (Figure 1) were not found to be underrepresented in these other cell types (Figure 2). These data suggest that cell-specific factors influence the degree of RISC association of individual miRNAs.

### RISC association of a microRNA predicts inhibitory potential

It has previously been reported that the ability of individual miRNAs to downregulate target mRNAs, including artificial indicator gene mRNAs, ‘does not correlate with miRNA concentration’ in the cell (11). We reasoned that this lack of correlation might, at least in part, reflect the fact that measurement of total miRNA levels does not accurately measure the level of a given miRNA that is in the RISC effector and, hence, capable of inhibiting mRNA expression. We therefore analysed the ability of several miRNAs that are expressed in 293 cells, and differentially RISC associated, to inhibit a transfected sensor construct containing three tandem miRNA target sites. These target sites were designed to contain mismatches to the miRNA at positions 10 and 11 to prevent cleavage and degradation by Ago2 RISC (14,15). In designing these sensor constructs, we sought to avoid sensors that would be regulated by more than one miRNA belonging to a particular ‘seed’ family, i.e. miRNAs that share nucleotide positions 2 through 8, which is the major determinant of miRNA target specificity (16). However, because miR-92a-3p, miR-92b-3p and miR-25 bear the same seed sequence, we predict that all three of these miRNAs would target the same miRNA sensor in this assay and sequencing reads specific for these three miRNAs were therefore pooled in this experiment.

**Figure 3.**
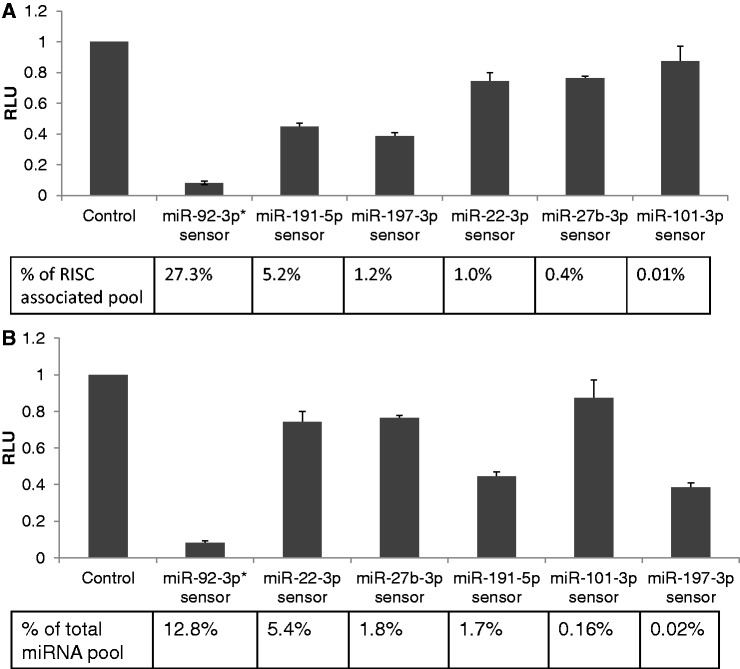
MiRNA inhibitory potential in 293 cells correlates with the RISC-associated miRNA level, but not with the total miRNA level. (**A**) Sensor constructs were generated by insertion of three copies of a predicted full-length miRNA target site, bearing a bulge opposite miRNA nucleotides 10 and 11, for miR-92a-3p, miR-191-5p, miR-197-3p, miR-22-3p, miR-27b-3p or miR-101-3p. The control construct lacked inserted target sites. Sensor constructs were transfected into 293 cells and cells were harvested 24 h later and RLuc and FLuc (internal control) activities determined. Data are expressed relative to the value for the control construct, which was set at 1. The observed level of inhibition was then plotted relative to the observed level of the individual miRNA analysed in the RISC-associated miRNA pool, given as a percentage. Values shown represent averages of three independent experiments, with s.d. indicated. (*)Note that the miR-92-3p sensor is predicted to detect not only miR-92a-3p but also miR-92b-3p and miR-25 and reads specific for these three miRNAs are therefore summed in this analysis. (**B**) Same experiment as in A but plotted in the order of the miRNA expression level observed in the total small RNA pool.

We introduced the miRNA sensor constructs into 293 cells and analysed the relative level of inhibition observed at 24 h post-transfection. The observed inhibition is plotted as a function of the level of the individual, endogenous miRNA or, in the case of the miR-92-3p sensor, the relevant miRNA family, detected in either RISC (Figure 3A) or the total miRNA pool (Figure 3B). As may be observed, there is a strong and significant (Pearson correlation *r* = −0.83, *P* < 0.05) negative correlation between the level of a given endogenous miRNA found in RISC and the level of sensor gene expression observed (Figure 3A). Conversely, plotting these same sensor gene data relative to the relative level of miRNA expression in the total miRNA population (Figure 3B) failed to reveal a significant correlation (Pearson correlation *r* = −0.64, *P* = 0.17). We note, however, that RISC association does not perfectly predict functionality in this assay, e.g. miR-191-5p appears to be a less effective inhibitor than miR-197-3p yet is found in RISC at levels that are ∼4-fold higher (Figure 3A). This discrepancy could be due to sequence-specific differences in the recovery of these miRNAs in the cDNA library used for deep sequencing, as previously reported (12,13) or could imply that miRNA function is also controlled by other factors, such as the prevalence of endogenous mRNA targets, and not just by the level of RISC association.

**Figure 4.**
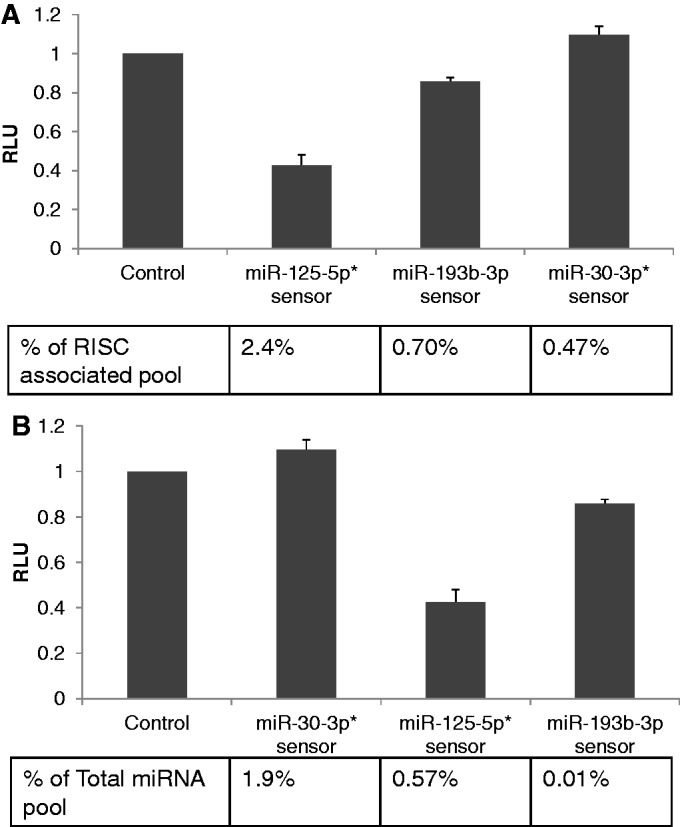
MiRNA inhibitory potential in A549 cells correlates with the RISC-associated miRNA level. (**A**) Sensor constructs were generated by the insertion of three copies of a predicted full-length miRNA target site, bearing a bulge opposite to miRNA nucleotides 10 and 11, for miR-125-5p, miR-193b-5p and miR-30-3p. The sensor constructs were transfected into A549 cells and cells were harvested 24 h later and RLuc and FLuc (internal control) activities determined. Data are expressed relative to the value for the control construct, which was set at 1. The observed level of inhibition was then plotted relative to the observed level of the individual miRNA analysed in the RISC-associated miRNA pool, given as a percentage. The values shown represent the average of three independent experiments, with s.d. indicated. (*)Note that the miR-125-5p sensor will detect both miR-125a-5p and miR-125b-5p while the miR-30-3p sensor will detect both miR-30a-3p and miR-30e-3p. The reads for these two sets of two miRNAs are therefore independently summed in this analysis. (**B**) Same experiment as A but plotted in the order of the miRNA expression level observed in the total small RNA pool.

To extend these data beyond the 293 cell line, we repeated this same analysis in the human A549 cell line using an entirely distinct set of differentially expressed endogenous miRNAs. Again, the sensor used for miR-30-3p is predicted to sense two endogenous miRNAs, the closely related miR-30a-3p and miR-30e-3p, both of which are equivalently underrepresented in RISC (Figure 2A, Supplementary Table S2), and reads specific to these two miRNAs are therefore pooled in this analysis. Similarly, the miR-125-5p sensor will detect both miR-125a-5p and miR-125b-5p, which again show a very similar level of RISC association in A549 cells (Figure 2A and Supplementary Table S2), and these two miRNAs are therefore summed. As shown in Figure 4, we again observed a correlation between the level of RISC association and the inhibitory potential for these miRNAs, while the level of expression in the total miRNA pool failed to predict the level of sensor gene inhibition, as also previously reported by others (11).

### Highly complementary mRNA targets can enhance microRNA association with RISC

**Figure 5.**
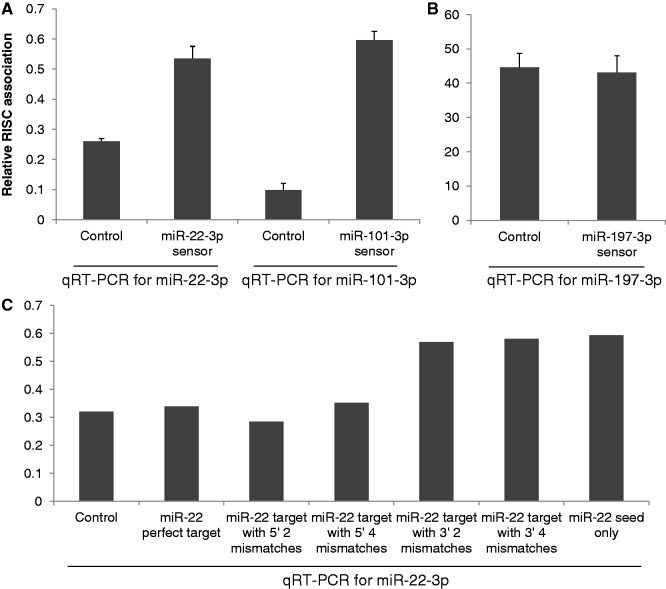
Overexpression of a highly complementary miRNA target can enhance RISC association. (**A**) 293 cells were transfected with sensor constructs containing three copies of a predicted full-length miRNA target site bearing a bulge opposite miRNA nucleotides 10 and 11, or a control construct containing no inserted targets. Three days post-transfection, cells were collected and total small RNAs (≤200 nt), and RISC-associated, RNAs isolated. The indicated qRT-PCR analysis of miR-22-3p and miR-101-3p expression levels was normalized to miR-138, which does not show differential RISC association. The observed contribution of each miRNA relative to the RISC-associated miRNA pool is given relative to its contribution to the total miRNA pool, which was set at 1.0. Average of three independent experiments with s.d. indicated. (**B**) Similar to A, except that miR-197, which is highly RISC-associated, was analysed. (**C**) Similar to A, except that the sensor constructs used contained three perfect, fully complementary targets for miR-22-3p; three targets bearing mismatches opposite miRNA 5′ positions 1 and 2, three targets bearing mismatches opposite miRNA 5′ positions 1,2,3 and 4, three targets bearing mismatches opposite miRNA 3′ positions 21 and 22; three targets bearing mismatches opposite miRNA 3′ positions 19, 20, 21 and 22; or three targets showing only seed homology (nt 1–8) to miR-22-3p. Total and RISC-associated miRNA levels were analysed by qRT-PCR at 72 h post-transfection and are plotted as indicated in A.

Previous work has suggested that the level of expression and degree of complementarity of target mRNAs can significantly affect the level of expression of specific miRNAs, though these reports have differed as to whether target mRNAs stabilize miRNAs (17,18) or destabilize (14,19–21) miRNAs. If dissociation from RISC represents an early step in the miRNA degradation pathway, we reasoned that overexpression of a highly complementary mRNA target might affect the rate of RISC dissociation of a specific miRNA. To test this hypothesis, we used some of the same sensor constructs analysed in Figure 3 and overexpressed the encoded sensor mRNAs specific for miR-22-3p, miR-101-3p and miR-197-3p in 293 cells. The level of these sensors used in this experiment was, however, 10-fold higher than used in the functional assays shown in Figure 3, and the effect on miRNA expression, as measured by qRT-PCR, was analysed at 72 h post-transfection, rather than the 24 h timepoint shown in Figure 3. To reiterate, these sensor mRNAs contain three tandem target sites for each miRNA that are perfectly complementary except for mismatches at miRNA positions 10 and 11. We note that similar miRNA targets have previously been reported to destabilize miRNAs in transfected cells (14), and we were therefore concerned that we would observe a substantial drop in the level of expression of the cognate miRNA in the total miRNA pool. However, this was not observed at 3 days after transfection (miR-22-3p levels were 1.03 ± 0.56 relative to control cells; miR-101-3p levels were at 0.98 ± 0.19; and miR-197-3p levels were 1.16 ± 0.47. Average of three experiments with s.d. indicated.). Analysis in parallel of the level of RISC-associated miRNAs did, however, reveal a significant and surprising difference relative to control cells. Specifically, miR-22-3p and miR-101-3p, both of which are normally weakly RISC associated in 293 cells (Figure 1), increased their RISC association by ∼2-fold and by ∼6-fold, respectively (Figure 5A). In contrast, in the case of miR-197-3p, which is constitutively highly RISC associated (Figure 1), expression of a similar cognate target mRNA species had no significant effect (Figure 5B).

To extend these data further, we also analysed similar sensor constructs containing a fully complementary miR-22-3p target or targets containing mismatches at 5′ positions 1 and 2, or 1, 2, 3 and 4, or mismatches at 3′ positions 19, 20, 21 and 22, or at only position 21 and 22. Note that the constructs with mismatches at position 1 and 2, or 1, 2, 3 and 4, lack full seed homology to miR-22 and therefore are predicted to be poor targets for miR-22 binding *in vivo*. We also tested an indicator construct containing three tandem miR-22-3p targets that had full complementarity to the miR-22-3p seed (nucleotides 1–8), but no other sequence homology. As shown in Figure 5C, we in fact did not see any effect on RISC association mediated by either the mRNA targets lacking full seed homology or, perhaps surprisingly, the fully complementary miR-22-3p target. In contrast, the two constructs bearing mismatches at positions 19–22, or only at 21 and 22, as well as the target that was only complementary to the miR-22-3p seed, all enhanced the RISC association of miR-22-3p by ∼2-fold. Therefore, our data indicate that specific mRNA targets can influence the RISC association of a given miRNA. Surprisingly, the effect was to enhance RISC association, not inhibit it.

### Level of 3′ tailing of microRNAs does not predict the degree of RISC association

Previous work has suggested that miRNA degradation occurs via the sequential tailing and trimming of the miRNA 3′ end (14,20,22), and it has also been reported that the stability of cellular miR-122 is selectively enhanced by the 3′ terminal addition of an adenosine (23). If the miRNA population in cells that is not bound by RISC represents a pool that is destined for degradation, then one would predict that 3′ tailing would be more common in the free miRNA pool than the RISC-associated miRNA pool. Analysis of the level of tailing of miRNAs by deep sequencing revealed readily detectable 3′ tailing of all the miRNAs expressed in 293 and A549 cells. However, the level of 3′ tailing varied widely, with >20% of all reads specific for miR-101-3p bearing 3′ tails while <0.3% of the reads specific for miR-22-3p were tailed. A summary of the level of tailing of selected human miRNAs in the total or RISC-associated small RNA pool is given in Supplementary Table S3, while an analysis of the sequence identity of tails seen on representative miRNA species is presented in Supplementary Table S4. As previously reported (24), these tails were quite short, ≤3 nt in length, and tended to be rich in A and T residues, although some C residues were also observed.

**Figure 6.**
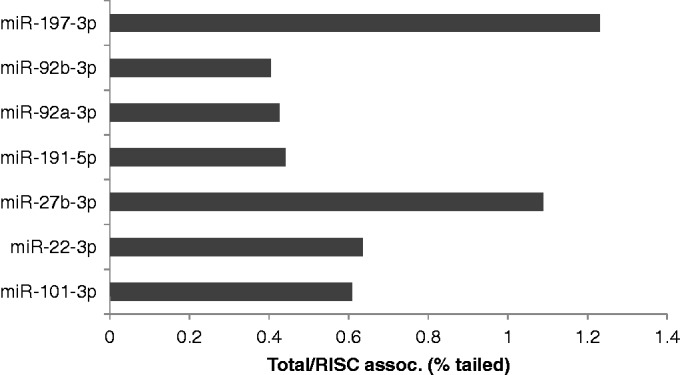
Level of RISC association of a miRNA is not predicted by the level of 3′ tailing. miRNAs bearing 3′ tails were identified by deep sequencing (Supplementary Tables S3 and S4), and the percentage of tailed miRNAs in the total small RNA and RISC-associated small RNA libraries was determined in 293 cells. These percentages are presented as a ratio of percentage tailed in Total vs. percentage tailed in the RISC-associated pool, with the miRNAs listed in descending order of RISC association. A similar, more extensive, analysis looking at all miRNAs expressed in 293 cells or A549 cells is presented in Supplementary Figure S3.

The key question we wished to address was whether the level of tailing, which is thought to be linked to miRNA degradation (14,20,22), is higher in the total miRNA pool than in the RISC-associated miRNA pool, thus implying that the former is enriched in miRNAs destined for degradation. To address this, we determined the percentage of the miRNA reads in the total and RISC-associated miRNA pools that were tailed and then determined the ratio of this percentage in the total miRNA pool to the percentage seen in the RISC-associated miRNA pool. If the total miRNA pool is indeed enriched for miRNAs destined for degradation, and hence presumably tailed, then this ratio should be >1. In Figure 6, we show a representative set of seven miRNAs that, in 293 cells, show either high (top of figure) or low (bottom of figure) RISC association, while all miRNAs contributing ≥0.1% of reads in either the total or RISC-associated miRNA pool in 293 and A549 cells are analysed in Supplementary Figure S3. As may be seen, we observed no consistent relationship between the relative level of RISC association and the relative level of 3′ tailing for miRNAs in the total versus RISC-associated miRNA pool, although our data did, surprisingly, suggest that RISC-associated miRNAs were in fact slightly more likely to bear a 3′ tail, at least in 293 cells (Supplementary Figure S3). Analysis of the sequences of the observed miRNA 3′ tails showed these to be similar in the total and RISC-associated miRNA pools (Supplementary Table S4). Nevertheless, we did observe that some tails were somewhat more prevalent in the total miRNA pool than in the RISC-associated miRNA pool. Specifically, a single non-templated ‘G’ as well as the dinucleotide tail ‘CC’ were all 2- to 3-fold more prevalent in the total miRNA pool than in the RISC-associated miRNA pool in both 293T cells and A549 cells (data not shown). However, we did not see any significant effect of a single 3′ terminal non-templated ‘A’ residue, as has been previously proposed (23). Overall, these data therefore argue that 3′ tailing has at most a modest effect on the level of RISC association of miRNAs, though individual 3′ tail sequences may be more functionally significant than 3′ tailing *per se*.

## DISCUSSION

Numerous articles have reported the conventional or, more recently, deep sequencing of miRNAs present in the total small RNA population in various cell types and tissues. An implicit assumption underlying these studies is that the total miRNA pool is an accurate reflection of the pool of miRNAs that is associated with the RISC effector. This assumption was based on the expectation that small RNAs, lacking the terminal protection mediated by covalent modifications or bound proteins, would be rapidly degraded by the abundant RNA exonucleases present in cells (2–5). Indeed, the full-length passenger strands released after loading of the miRNA strand into RISC are often not detected at significant levels in the total small RNA pool, thus suggesting that these similar ∼22-nt RNAs are indeed highly labile. However, recent results have challenged the expectation that miRNAs that are present in the total miRNA pool are an accurate measure of the RISC-associated miRNA population. In particular, Janas *et al.* (6) reported that total mature miRNAs in HeLa cells were present at an ∼13-fold excess over the total number of Ago proteins, while Stalder *et al.* (7) reported that a major fraction of the miRNA pool was not Ago-associated. These recent data are consistent with earlier data obtained in Drosophila showing that a high percentage of the small interfering RNAs generated in this invertebrate organism on viral infection are not, in fact, associated with RISC (25,26).

We reasoned that if a large proportion of the miRNA population is indeed not RISC associated, then the relative level of RISC association of individual miRNAs might be regulated in different cell types. Moreover, if miRNAs that are not associated with an Ago protein are unable to repress mRNA function, as one would expect based on our current understanding of RISC function (16), then the degree of RISC association might influence the inhibitory potential of specific miRNAs.

Here, we present data strongly arguing that both these hypotheses are correct. By deep sequencing or qRT-PCR analysis of the relative expression of endogenous human miRNAs in either the total or RISC-associated miRNA pool, we observed large variations in the level of RISC association, with overall differences of >100-fold between the most highly RISC-associated and least RISC-associated miRNAs in all cell types examined (Figures 1 and 2 and Supplementary Tables S1 and S2). Moreover, this discrepancy was observed using different monoclonal antibodies to isolate RISC (Figure 1A and B), in several different cell lines (Figures 1 and 2), was not due to inefficient recovery of cellular Ago proteins (Supplementary Figure S2) and was seen regardless of whether deep sequencing or qRT-PCR was used to measure miRNA expression (Figure 1). While it has been previously reported that miRNAs with low G-C content can be selectively lost during small RNA isolation using TRIzol from small numbers of cells (27), we note that the step where these small RNAs were lost, during the precipitation step after extraction, does not form part of our isolation protocol, which instead used the mirVana kit shown by these same authors to recover all small RNAs stoichiometrically (27). Moreover, we did not start with small numbers of cells and the level of RISC association of miRNAs was not predicted by their level of G and C residues. For example, the 21 miRNAs that are >2-fold overrepresented in RISC in A549 cells show an average G+C content of 48%, while the 18 miRNAs that are >2-fold underrepresented in RISC show a G+C content of 49%. We are therefore confident that all possible artifactual explanations for this result have been addressed. The finding that miRNAs with a low G-C content are neither under- nor overrepresented in RISC also argues that base composition *per se* is not a factor in whether a miRNA is retained in RISC.

As noted above, if the level of miRNA-association with RISC is indeed highly variable, then one would predict that the inhibitory potential of a given miRNA would correlate with the level of a given miRNA in RISC, not by its representation in the total small RNA pool. Indeed, previous work has demonstrated that total miRNA expression levels only weakly predict miRNA function (11). To address this question, we selected several miRNA that showed substantial differences in their endogenous expression levels in the total and RISC-associated miRNA pools and examined their inhibitory potential in either 293 or A549 cells using artificial indicator constructs (Figures 3 and 4). In 293 cells, we observed that RISC association indeed predicted the level of repression observed (*P* < 0.05), while expression in the total miRNA pool did not (Figure 3), and very similar data were also obtained in A549 cells (Figure 4). These data therefore argue that future efforts to predict miRNA function in other settings would be more likely to be accurate if they were based on an analysis of RISC-associated, rather than total, miRNA expression. Nevertheless, it is possible that RISC function is also controlled by other factors, and it is well established that current small RNA deep sequencing protocols can over- or underestimate the actual level of expression of a given miRNA, whether in RISC or in the total RNA pool (12,13).

While we observed significant variation in the level of RISC association of miRNAs in all the cell types examined, we noticed that individual miRNAs sometimes differed in their level of RISC association in different cell types. For example, miR-92b-3p is ∼6-fold more RISC associated than the average miRNA in 293 cells yet only ∼0.86-fold as RISC associated as the average miRNA in A549 cells (Supplementary Tables S1 and S2). Similarly, miR-22-3p is ∼5-fold less RISC associated than the average miRNA in 293 cells but is RISC associated at the average level (0.94-fold) in A549 cells. These data show that the level of RISC association can vary substantially for a given miRNA in different cell types. One possible source of this variability is that the mRNA transcriptome, and hence the available mRNA target population, is likely to be quite different in different cell types. To examine whether the availability of different targets can influence RISC association, we therefore overexpressed various different mRNAs bearing three artificial miRNA target sites and asked whether these would exert an effect on the observed level of RISC association. As shown in Figure 5, we in fact observed that several different target mRNAs bearing sites with full seed homology to the miRNA enhanced the RISC association of weakly, but not strongly, RISC-associated miRNAs. An exception arose in the case of targets that were perfectly complementary to the miRNA along their entire length. This may be due to the fact that this mRNA target was highly repressed in these transfected cells, most likely because of efficient cleavage by Ago2-containing RISCs, and may therefore be present at very low levels. In contrast, mRNA targets that were complementary along their entire length, except at residues 10 and 11 where cleavage by Ago2 would occur (14,15), or that retained full miRNA seed homology but bore limited homology to the miRNA 3′ end, did enhance the RISC association of weakly RISC-associated miRNAs (Figure 5). However, expression in the total miRNA pool was essentially unaffected. It is interesting to contrast this *in vivo* result with recent *in vitro* data suggesting that highly complementary mRNA targets can promote the release of Ago2 from miRNAs (28). In this analysis, RISC unloading *in vitro* was enhanced by mismatches at the 5′, seed end of miRNA targets but prevented by mismatches at the 3′ end. However, the targets bearing 5′ mismatches in the seed were not found to effectively induce RISC unloading *in vivo*, most probably because lack of seed homology inhibited miRNA–mRNA interactions (28). While our data do not therefore fully agree with this reported *in vitro* analysis, we do agree that mismatches to the 3′ end of a target can prevent the target-mediated unloading of a miRNA from RISC and can even enhance the stability of RISC association.

A difficult issue to address is that sensor constructs can apparently not only detect miRNA function (Figures 3 and 4) but also affect the level of of an miRNA (Figure 5). To try to circumvent this problem, we used a 10-fold higher level of the miRNA sensors in the experiments designed to address whether target mRNA can influence RISC loading (Figure 5) and performed these analyses at 72 h post-transfection, rather than the 24 h timepoint used in assays for miRNA function (Figures 3 and 4). Moreover, we note that our data suggest that highly complementary miRNA targets can enhance the RISC association of weakly RISC-associated miRNAs that our functional data indicate are also only weakly active (Figure 3). Therefore, if low levels of these same miRNA sensors do enhance RISC association within 24 h of transfection, when miRNA inhibitory activity was measured (Figure 3), then our data would, in fact, underestimate the defect in target mRNA repression caused by the weak RISC association of these miRNAs.

One final issue examined in this manuscript relates to the question of whether 3′ tailing of miRNAs, which has been proposed to initiate miRNA degradation (14,20,22), predicts the level of RISC association. Our data (Figure 6 and Supplementary Figure S2) suggest that it does not, as we detected comparable levels of miRNA tailing in both the RISC-bound and total miRNA pools. Therefore, tailing can apparently occur before a miRNA is loaded into RISC, or while a miRNA is RISC associated, and tailing *per se* does not appear to promote RISC unloading. Nevertheless, we did note that some specific miRNA 3′ tails, particularly ‘G’ and ‘CC’, were somewhat more prevalent in the total miRNA pool in both 293T and A549 cells, so specific 3′ tails may exert a modest effect on RISC loading and unloading.

While our data clearly indicate that the level of miRNA association with RISC can vary widely, and suggest that the level of miRNA RISC association is predictive of inhibitory potential, we do not currently understand how RISC association is regulated or where the stable, non–RISC-associated miRNAs are located, though others have suggested that mature miRNAs that are not loaded into RISC are bound to cellular mRNAs (6). However, our data are consistent with the hypothesis that the level of RISC association of miRNAs is influenced by the available repertoire of mRNA targets, as also previously proposed by others (28), although our data suggest that target mRNAs can enhance, not only inhibit, RISC association (Figure 5). We believe our data suggest that differential RISC association primarily reflects differences in the rate of RISC unloading, rather than loading. In particular, we note that the miRNA passenger strands for the endogenous miRNAs analysed in Figures 3 and 4 are found at only very low levels, consistent with their degradation during unloading, and moreover that the level of RISC association of the miRNA guide strand is not predictive of the level of RISC association of the passenger strand, thus suggesting that retention in RISC is regulated after loading has occurred (Supplementary Table S5). Regardless, our data clearly indicate that the level of RISC association of individual miRNAs is differentially regulated in different cell types, most probably due to global differences in the mRNA transcriptome and hence miRNA target site repertoire in different cell types, and suggest that this differential regulation is as important, or more important, than the absolute expression level of the miRNA in predicting miRNA inhibitory potential.

## ACCESSION NUMBERS

RNA sequencing data will be deposited in the Gene Expression Omnibus (GEO) once this article has been accepted for publication.

## SUPPLEMENTARY DATA

Supplementary Data are available at NAR Online.

## FUNDING

National Institutes of Health [R01-AI067968, T32-CA009111, T32-AI007392.]; Duke Center for AIDS Research (CFAR) small grant award from the National Institutes of Health [P30-AI064518 to R.L.S.]. Funding for open access charge: R01-AI067968.

*Conflict of interest statement*. None declared.

## Supplementary Material

Supplementary Data
