# Supplementary material for: Differential RISC association of endogenous human microRNAs predicts their inhibitory potential
Source: Nucleic Acids Res. 2014 Jan 23;42(7):4629–39. doi: 10.1093/nar/gkt1393 (PMC3985621; doi:10.1093/nar/gkt1393)
Supplement: Supplementary Data [file supp_42_7_4629__index.html]

Differential RISC association of endogenous human microRNAs predicts their inhibitory potential — Differential RISC association of endogenous human microRNAs predicts their inhibitory potential — Supplementary Data 

# Differential RISC association of endogenous human microRNAs predicts their inhibitory potential

## Supplementary Data

files

**Files in this Data Supplement:**

- Supplementary Data - pdf file
