# Supplementary material for: Differential RISC association of endogenous human microRNAs predicts their inhibitory potential
Source: Nucleic Acids Res. 2014 Jan 23;42(7):4629–39. doi: 10.1093/nar/gkt1393 (PMC3985621; doi:10.1093/nar/gkt1393)
Supplement: Supplementary Data [file supp_gkt1393_nar-03404-y-2013-File008.pdf]

Figure S1. The level of expression of miRNAs present in RISC in 293 cells was determined by immunoprecipitation using the distinct pan-Ago monoclonal antibodies 2A8 and ab57113, followed by deep sequencing. This graph, derived as described in Figure 1, shows that there is a tight correlation between the level of expression detected using either antibody.

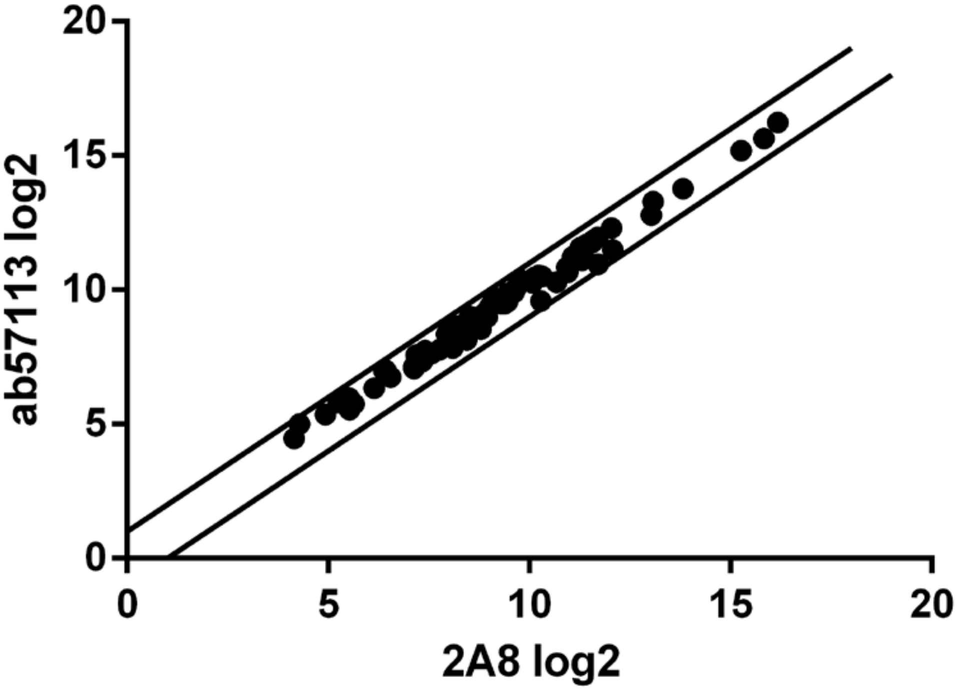

Figure S2. The level of total Ago protein immunoprecipitated in this study was determined using Ab57113 for immunoprecipitation, as described below, and the 2A8 antibody for Western blot analysis. This Western shows that the vast majority of expressed Ago protein, and hence the associated miRNAs, are solubilized and recovered using the described IP method.

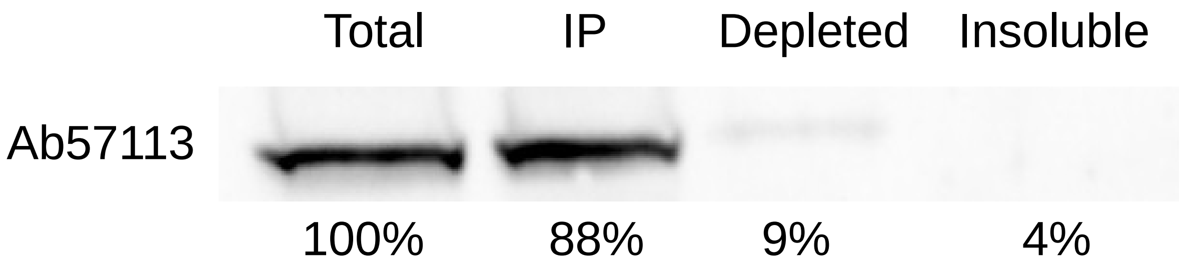

Methods

This Ago immunoprecipitation was performed as described in Materials and Methods for miRNA recovery. Briefly, $5x10^7$ cells were lysed in lysis buffer and then centrifuged to remove any particulate matter. The resultant pellet was then placed in loading dye (2% SDS, 10% glycerol, 0.5% bromophenol blue, 0.06 M TRIS-HCl pH 6.8 and 5% β-mercaptoethanol) and incubated at 95°C for 30 minutes. A portion of this lysate was retained as “total lysate” and the remainder incubated with protein G beads pre-loaded with the pan-Ago antibody ab57113 at 4°C overnight on a rotator. Next day, the protein G beads were isolated by centrifugation and the depleted supernatant removed. Equal portions of total lysate, depleted lysate and protein G beads (IP) were then mixed with loading dye or 2x loading dye and incubated at 95°C for 5 minutes. Equal aliquots of total lysate, depleted lysate, protein G beads (IP) and insoluble fraction were then loaded onto a 4-20% TRIS-HCl gel and subjected to electrophoresis. The gel was then used for a Western blot using the 2A8 pan-Ago monoclonal antibody (Diagenode) and an anti-mouse secondary antibody (Sigma A9044), visualized using ECL according to the manufacturer’s directions (Advansta, Western Bright K-12043-D20) and quantified using a Syngene GBox with background subtracted.

Figure S3. Effect of 3' tailing on the level of Ago binding of microRNAs. This figure lists all miRNAs that contribute >0.1% of the total or RISC-associated miRNA pool, in descending order of their level of RISC association in 293 cells (A) or A549 cells (B). The x-axis presents the ratio of the percentage of tailed miRNAs observed in the Total vs. RISC-associated miRNA pool.

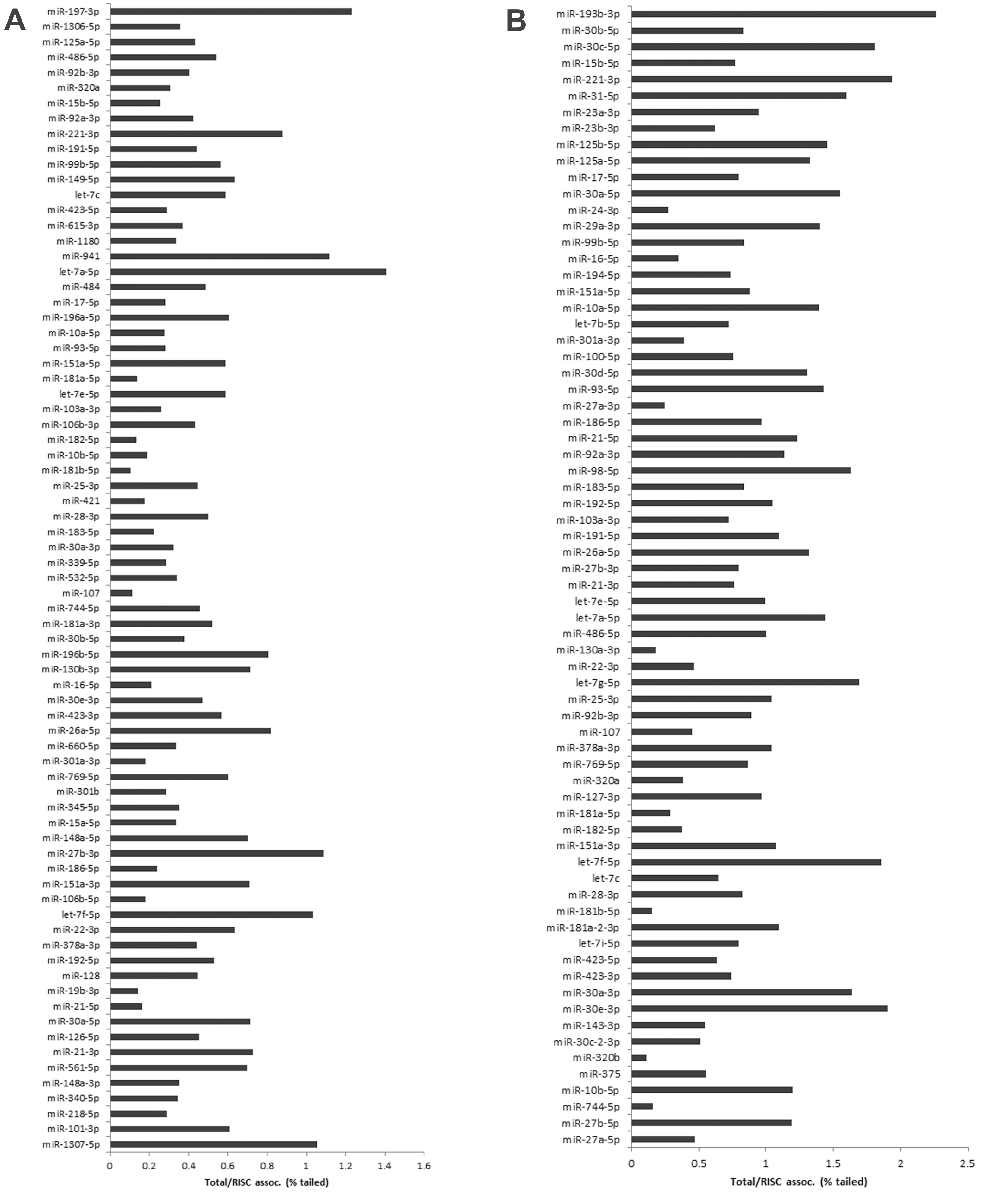

Table S1. Level of RISC association of endogenous miRNAs expressed in 293 cells.

293/2A8	% Total miRNA pool	% RISC-assoc. pool	Ratio
miR-197-3p	0.02%	1.21%	59.33
miR-1306-5p	0.00%	0.14%	59.01
miR-125a-5p	0.10%	0.92%	9.34
miR-486-5p	0.07%	0.45%	6.04
miR-92b-3p	0.51%	3.04%	5.94
miR-320a	0.15%	0.72%	4.80
miR-15b-5p	0.05%	0.19%	3.61
miR-92a-3p	5.98%	21.10%	3.53
miR-221-3p	0.13%	0.41%	3.10
miR-191-5p	1.71%	5.23%	3.06
miR-99b-5p	0.37%	1.10%	2.97
miR-149-5p	0.06%	0.16%	2.88
let-7c	0.06%	0.17%	2.83
miR-423-5p	0.58%	1.56%	2.68
miR-615-3p	0.05%	0.13%	2.65
miR-1180	0.05%	0.12%	2.60
miR-941	0.19%	0.46%	2.42
let-7a-5p	0.58%	0.90%	1.55
miR-484	0.10%	0.13%	1.36
miR-17-5p	0.09%	0.11%	1.27
miR-196a-5p	0.08%	0.10%	1.26
miR-10a-5p	23.68%	26.75%	1.13
miR-93-5p	0.84%	0.94%	1.11
miR-151a-5p	0.24%	0.26%	1.06
miR-181a-5p	0.94%	0.98%	1.04
let-7e-5p	0.22%	0.23%	1.02
miR-103a-3p	1.21%	1.20%	0.99
miR-106b-3p	0.11%	0.10%	0.94
miR-182-5p	1.64%	1.53%	0.93
miR-10b-5p	17.41%	14.26%	0.82
miR-181b-5p	0.32%	0.25%	0.79
miR-25-3p	4.29%	3.14%	0.73
miR-421	0.26%	0.18%	0.70
miR-28-3p	1.06%	0.71%	0.67
miR-183-5p	0.15%	0.10%	0.66
miR-30a-3p	0.22%	0.14%	0.64
miR-339-5p	0.12%	0.07%	0.60
miR-532-5p	0.32%	0.18%	0.58
miR-107	0.39%	0.21%	0.55
miR-744-5p	1.11%	0.59%	0.53
miR-181a-3p	0.24%	0.13%	0.53
miR-30b-5p	0.12%	0.06%	0.50
miR-196b-5p	0.48%	0.21%	0.45
miR-130b-3p	0.22%	0.10%	0.44
miR-16-5p	2.75%	1.12%	0.41
miR-30e-3p	0.25%	0.10%	0.41
miR-423-3p	2.24%	0.85%	0.38
miR-26a-5p	2.12%	0.79%	0.37
miR-660-5p	0.15%	0.05%	0.34
miR-301a-3p	0.41%	0.13%	0.33
miR-769-5p	1.26%	0.39%	0.31
miR-301b	0.11%	0.03%	0.29
miR-345-5p	0.32%	0.09%	0.28
miR-15a-5p	0.11%	0.03%	0.28
miR-148a-5p	0.19%	0.05%	0.27
miR-27b-3p	1.81%	0.44%	0.24
miR-186-5p	1.06%	0.24%	0.23
miR-151a-3p	0.61%	0.13%	0.22
miR-106b-5p	0.16%	0.03%	0.21
let-7f-5p	1.50%	0.32%	0.21
miR-22-3p	5.38%	1.00%	0.19
miR-378a-3p	0.90%	0.17%	0.18
miR-192-5p	1.69%	0.28%	0.17
miR-128	0.48%	0.08%	0.16
miR-19b-3p	0.11%	0.02%	0.15
miR-21-5p	0.44%	0.06%	0.14
miR-378c	0.13%	0.02%	0.13
miR-30a-5p	0.14%	0.02%	0.12
miR-126-5p	0.23%	0.03%	0.11
miR-21-3p	0.16%	0.01%	0.09
miR-561-5p	0.21%	0.02%	0.08
miR-148a-3p	3.68%	0.30%	0.08
miR-340-5p	0.66%	0.05%	0.08
miR-218-5p	0.13%	0.01%	0.06
miR-101-3p	0.16%	0.01%	0.04
miR-1307-5p	0.34%	0.01%	0.03

This table lists all miRNAs expressed in 293 cells at >0.1% of the total or RISC-associated miRNA pool, 76 in total, in order of their level of RISC association, with the most highly RISC-associated listed first. In particular, this table presents the level of expression of each miRNA as a percentage of the total miRNA pool (column 2) and RISC-associated miRNA pool (column 3) and then gives the ratio of these two numbers (column 4). The miRNAs functionally analyzed in Fig. 3 are shown in yellow.

Table S2. Level of RISC association of endogenous miRNAs expressed in A549 cells.

A549/ab57113	% Total miRNA pool	% RISC-assoc. pool	Ratio
miR-193b-3p	0.01%	0.70%	64.76
miR-30b-5p	0.02%	0.22%	11.56
miR-30c-5p	0.02%	0.17%	8.47
miR-15b-5p	0.03%	0.26%	7.75
miR-221-3p	0.14%	0.93%	6.61
miR-31-5p	0.04%	0.20%	5.53
miR-23a-3p	0.06%	0.29%	4.62
miR-23b-3p	0.07%	0.31%	4.55
miR-125b-5p	0.05%	0.22%	4.54
miR-125a-5p	0.52%	2.15%	4.13
miR-17-5p	0.03%	0.11%	3.98
miR-30a-5p	0.12%	0.38%	3.20
miR-24-3p	0.05%	0.15%	3.20
miR-29a-3p	0.06%	0.16%	2.77
miR-99b-5p	0.69%	1.89%	2.74
miR-16-5p	0.66%	1.72%	2.60
miR-194-5p	0.12%	0.30%	2.44
miR-151a-5p	0.35%	0.83%	2.34
miR-10a-5p	2.88%	6.60%	2.29
let-7b-5p	0.19%	0.39%	2.06
miR-301a-3p	0.05%	0.10%	2.02
miR-100-5p	0.23%	0.45%	1.95
miR-30d-5p	0.06%	0.11%	1.94
miR-93-5p	0.31%	0.57%	1.82
miR-27a-3p	0.22%	0.39%	1.78
miR-186-5p	0.11%	0.19%	1.75
miR-21-5p	3.11%	5.27%	1.69
miR-92a-3p	4.03%	6.66%	1.65
miR-98-5p	0.11%	0.16%	1.47
miR-183-5p	0.08%	0.11%	1.42
miR-192-5p	8.29%	11.72%	1.41
miR-103a-3p	0.62%	0.86%	1.40
miR-191-5p	3.48%	4.56%	1.31
miR-26a-5p	1.08%	1.39%	1.29
miR-27b-3p	10.94%	14.04%	1.28
miR-21-3p	2.01%	2.31%	1.15
let-7e-5p	0.63%	0.68%	1.09
let-7a-5p	2.49%	2.58%	1.04
miR-486-5p	0.22%	0.23%	1.03
miR-130a-3p	0.16%	0.15%	0.97
miR-22-3p	7.83%	7.38%	0.94
let-7g-5p	0.23%	0.21%	0.93
miR-25-3p	1.74%	1.59%	0.91
miR-92b-3p	1.52%	1.30%	0.86
miR-107	0.17%	0.15%	0.85
miR-378a-3p	0.16%	0.13%	0.79
miR-769-5p	0.22%	0.17%	0.75
miR-320a	0.53%	0.40%	0.75
miR-127-3p	0.30%	0.22%	0.72
miR-181a-5p	2.99%	2.12%	0.71
miR-182-5p	4.13%	2.89%	0.70
miR-151a-3p	0.64%	0.32%	0.50
let-7f-5p	3.34%	1.62%	0.48
let-7c	0.18%	0.09%	0.48
miR-28-3p	2.98%	1.40%	0.47
miR-181b-5p	0.50%	0.22%	0.44
miR-181a-2-3p	0.53%	0.23%	0.43
let-7i-5p	6.63%	2.50%	0.38
miR-423-5p	0.64%	0.21%	0.34
miR-423-3p	0.86%	0.25%	0.30
miR-30a-3p	1.57%	0.41%	0.26
miR-30e-3p	0.30%	0.06%	0.19
miR-143-3p	0.24%	0.04%	0.18
miR-30c-2-3p	0.13%	0.02%	0.18
miR-320b	0.39%	0.06%	0.16
miR-375	0.10%	0.01%	0.14
miR-10b-5p	13.00%	1.73%	0.13
miR-744-5p	0.43%	0.05%	0.12
miR-27b-5p	0.94%	0.10%	0.10
miR-27a-5p	0.15%	0.01%	0.04

Identical to Table S1, except that these data are derived from A549 cells. MiRNAs highlighted in yellow were functionally analyzed in Fig. 4.

Table S3. Variable levels of 3' tailing of endogenous miRNAs in 293 cells.

**miRNA**	**Total miRNA pool**			**RISC-associated miRNA pool**		
	**Mature Reads**	**Tailed Reads**	**% Tailed**	**Mature Reads**	**Tailed Reads**	**% Tailed**
**miR-101-3p**	18,992	7,600	28.58	1,113	937	45.71
**miR-191-5p**	206,793	2,082	1.00	703,602	12,176	1.70
**miR-197-3p**	2,471	60	2.37	100,104	1,774	1.74
**miR-22-3p**	652,330	1,368	0.21	167,484	489	0.29
**miR-27b-3p**	218,903	19,974	8.36	74,298	5,149	6.48
**miR-92a-3p**	724,973	96,462	11.74	2,561,703	912,755	26.27

This table shows the number of total mature miRNA reads obtained for each of the indicated human miRNAs in either the total or RISC-associated miRNA pool, and the number of these reads that show 3' tails. These reads are then also given as a percentage of the total reads in each miRNA pool.

Table S4. Sequence of tails observed in selected miRNAs in 293 cells.

miR-92a-3p Total

Tailed Read Sequence	Number of Reads
**TATTGCACTTGTCCCGGCCTGTUGAGUU**	**Hairpin sequence**
TATTGCACTTGTCCCGGCCTGTAA	24,060
TATTGCACTTGTCCCGGCCTGTA	23,590
TATTGCACTTGTCCCGGCCTGTAT	15,725
TATTGCACTTGTCCCGGCCTGTC	6,741
TATTGCACTTGTCCCGGCCTGTCA	5,754
TATTGCACTTGTCCCGGCCTGTTT	3,645
TATTGCACTTGTCCCGGCCTGTCT	3,545
TATTGCACTTGTCCCGGCCTGTAG	2,540
TATTGCACTTGTCCCGGCCTGTAC	2,056
TATTGCACTTGTCCCGGCCTGTTA	1,416

miR-197-3p RISC-associated

Tailed Read Sequence	Number of Reads
**TTCACCACCTTCTCCACCCAGCATGGCC**	**Hairpin sequence**
TTCACCACCTTCTCCACCCAGCT	779
TTCACCACCTTCTCCACCCAGCC	410
TTCACCACCTTCTCCACCCAGCAA	105
TTCACCACCTTCTCCACCCAGCCT	52
TTCACCACCTTCTCCACCCAGCTT	39
TTCACCACCTTCTCCACCCAGCAAT	37
TTCACCACCTTCTCCACCCAGCG	32
TTCACCACCTTCTCCACCCAGCATT	28
TTCACCACCTTCTCCACCCAGCAAA	26
TTCACCACCTTCTCCACCCAGCCA	21

miR-92a-3p RISC-associated

Tailed Read Sequence	Number of Reads
**TATTGCACTTGTCCCGGCCTGTUGAGUU**	**Hairpin sequence**
TATTGCACTTGTCCCGGCCTGTAA	261,409
TATTGCACTTGTCCCGGCCTGTAT	161,291
TATTGCACTTGTCCCGGCCTGTA	140,928
TATTGCACTTGTCCCGGCCTGTCA	61,999
TATTGCACTTGTCCCGGCCTGTC	40,118
TATTGCACTTGTCCCGGCCTGTCT	35,852
TATTGCACTTGTCCCGGCCTGTAC	26,861
TATTGCACTTGTCCCGGCCTGTTT	26,179
TATTGCACTTGTCCCGGCCTGTAAT	22,523
TATTGCACTTGTCCCGGCCTGTAG	22,470

miR-197-3p Total

Tailed Read Sequence	Number of Reads
**TTCACCACCTTCTCCACCCAGCATGGCC**	**Hairpin sequence**
TTCACCACCTTCTCCACCCAGCT	32
TTCACCACCTTCTCCACCCAGCC	12

miR-22-3p Total

Tailed Read Sequence	Number of Reads
**AAGCTGCCAGTTGAAGAACTGTTCCCT**	**Hairpin sequence**
AAGCTGCCAGTTGAAGAACTGTA	1,087
AAGCTGCCAGTTGAAGAACTGTC	236
AAGCTGCCAGTTGAAGAACTGTAA	14
AAGCTGCCAGTTGAAGAACTGTTA	10
AAGCTGCCAGTTGAAGAACTGTG	5
AAGCTGCCAGTTGAAGAACTGTAT	5

miR-22-3p RISC-associated

Tailed Read Sequence	Number of Reads
**AAGCTGCCAGTTGAAGAACTGTTCCCT**	**Hairpin sequence**
AAGCTGCCAGTTGAAGAACTGTA	383
AAGCTGCCAGTTGAAGAACTGTC	77
AAGCTGCCAGTTGAAGAACTGTAA	8
AAGCTGCCAGTTGAAGAACTGTTA	5

miR-101-3p Total

Tailed Read Sequence	Number of Reads
**TACAGTACTGTGATAACTGAAGGAUG**	**Hairpin sequence**
TACAGTACTGTGATAACTGAAT	4,005
TACAGTACTGTGATAACTGAAA	2,187
TACAGTACTGTGATAACTGAAGT	715
TACAGTACTGTGATAACTGAAC	408
TACAGTACTGTGATAACTGAAGC	169
TACAGTACTGTGATAACTGAAAA	28
TACAGTACTGTGATAACTGAATT	23
TACAGTACTGTGATAACTGAAAC	20
TACAGTACTGTGATAACTGAAAT	15
TACAGTACTGTGATAACTGAACT	9

miR-101-3p RISC-associated

Tailed Read Sequence	Number of Reads
**TACAGTACTGTGATAACTGAAGGAUG**	**Hairpin sequence**
TACAGTACTGTGATAACTGAAT	404
TACAGTACTGTGATAACTGAAGT	220
TACAGTACTGTGATAACTGAAA	188
TACAGTACTGTGATAACTGAAC	51
TACAGTACTGTGATAACTGAAGC	36
TACAGTACTGTGATAACTGAAAA	10
TACAGTACTGTGATAACTGAATT	9

This table first presents the sequence of the mature miRNA, and predicted pri-miRNA stem extension, for the human miRNAs miR-92a-3p, miR-197-3p, miR-22-3p and miR-101-3p. It then shows all 3' tailed versions recovered that represent >10% of the total with a minimum of 5 reads, listed by read number observed.

Table S5. The level of RISC-association of miRNA guide strands does not predict the level of passenger strand association with RISC.

	Total Reads	% Total miRNA pool	RISC assoc. Reads	% RISC assoc. pool	Ratio
**293 cells**					
**miR-197-5p**	7	<0.001%	171	0.00%	18
**miR-197-3p**	2,471	0.02%	203,190	1.21%	59
**miR-92a-5p**	942	0.01%	1,923	0.01%	1.5
**miR-92a-3p**	724,973	5.98%	3,543,926	21.10%	3.5
**miR-22-5p**	44	<0.001%	133	0.00%	2.2
**miR-22-3p**	652,330	5.38%	168,281	1.00%	0.19
**miR-101-5p**	191	0.00%	17	<0.001%	0.064
**miR-101-3p**	18,992	0.16%	1,084	0.01%	0.041
**miR-27b-5p**	3,702	0.03%	1,066	0.01%	0.29
**miR-27b-3p**	218,903	1.81%	73,306	0.44%	0.33
**A549 cells**					
**miR-125a-5p**	57,612	0.52%	223,582	2.15%	3.9
**miR-125a-3p**	2,160	0.02%	1,618	0.02%	0.75
**miR-193b-5p**	197	<0.001%	826	0.01%	4.2
**miR-193b-3p**	1,195	0.01%	72,673	0.70%	61
**miR-30a-5p**	13,170	0.12%	39,551	0.38%	3
**miR-30a-3p**	173,817	1.57%	42,575	0.41%	0.24

This table presents the number of miRNA reads in the total and RISC-associated miRNA pool for both the guide and passenger strands of the endogenous miRNAs functionally analyzed in Figs. 4 and 5. These passenger strands are not listed in Tables S1 and S2, which only list miRNAs that represent >0.1% of the total or RISC-associated miRNA, with the exception of miR-30a-5p.
